# Crystallographic anisotropy of the resistivity size effect in single crystal tungsten nanowires

**DOI:** 10.1038/srep02591

**Published:** 2013-09-05

**Authors:** Dooho Choi, Matthew Moneck, Xuan Liu, Soong Ju Oh, Cherie R. Kagan, Kevin R. Coffey, Katayun Barmak

**Affiliations:** 1 Department of Materials Science and Engineering, Carnegie Mellon University, 5000 Forbes Avenue, Pittsburgh, Pennsylvania 15213, USA; 2 Korea Railroad Research Institute, 360-1 Woulam, Uiwang, Kyunggi 437-757, South Korea; 3 Department of Electrical and Computer Engineering, Carnegie Mellon University, 5000 Forbes Avenue, Pittsburgh, Pennsylvania 15213, USA; 4 Department of Materials Science and Engineering, University of Pennsylvania, 3451 Walnut Street, Philadelphia, Pennsylvania 19104, USA; 5 Department of Electrical and Systems Engineering, University of Pennsylvania, 3451 Walnut Street, Philadelphia, Pennsylvania 19104, USA; 6 Department of Materials Science and Engineering, University of Central Florida, 4000 Central Florida Boulevard, Orlando, Florida 32816, USA; 7 Department of Applied Physics and Applied Mathematics, Columbia University, 500 West 120^th^ Street, New York, New York 10027, USA

## Abstract

This work demonstrates an anisotropic increase in resistivity with decreasing width in single crystal tungsten (W) nanowires having a height of 21 nm. Nanowire-widths were in the range of 15–451 nm, with the anisotropy observed for widths below 50 nm. The longitudinal directions of the nanowires coincided with the <100>, <110> and <111> orientations of the body centered cubic phase of W. The resistivity increase was observed to be minimized for the <111>-oriented single crystal nanowires, exhibiting a factor of two lower increase in resistivity at a width of ~15 nm, relative to the thin film resistivity (i.e., an infinitely wide wire). The observed anisotropy is attributed to crystallographic anisotropy of the Fermi velocity and the resultant anisotropy of the electron mean free path in W, and underscores the critical role of crystallographic orientation in nanoscale metallic conduction.

The increase in resistivity with decreasing conductor dimension, termed the resistivity size effect, was first observed in 1901^1^, and the basic physics of the effect was understood by the mid-twentieth century^2^,^3^,^4^. However, the phenomenon has seen a recent resurgence of interest as a result of the severe resistivity increase observed in the nanoscale Cu wires that serve as interconnects in semiconductor devices^5^,^6^,^7^,^8^,^9^,^10^. This study extends the physics of the size effect to consider its potential crystallographic anisotropy and provides experimental verification of this anisotropy for the case of single crystal tungsten nanowires.

The resistivity size effect is typically attributed to the momentum loss of carriers along the axis of the conductors due to surface scattering and grain boundary scattering^11^. The two most widely used physical models for these scattering mechanisms are the Fuchs-Sondheimer (FS) surface scattering model, incorporating a specularity parameter, *p*, in the range of 0–1 for diffuse vs. specular scattering from surfaces^2^,^3^, and the Mayadas-Shatzkes (MS) grain boundary scattering model, incorporating a reflection coefficient, *R*, in the range of 0–1 for scattering from grain boundaries^4^. These models, based on the free electron theory, consider isotropic scattering processes and predict reduced resistivity size effect with decreasing electron mean free path. However, anisotropic carrier properties, e.g., in case of transition metals as suggested by the complex shape of the Fermi surface, may give rise to anisotropic scattering processes such that electrical resistivity of a sufficiently small conductor becomes affected by the crystallographic orientation of the structure.

In this study, the anisotropy of the resistivity size effect was demonstrated by using single crystal body centered cubic (bcc) W nanowires with their longitudinal axis coinciding with the <100>, <110> and <111> crystallographic directions. The wire-widths were in the range of 15–451 nm, with the anisotropy of the size effect observed for wire-widths below ~50 nm, i.e., at widths comparable to the directionally averaged electron mean free path of 19.1 nm for W.^12^ A reduction in incremental resistivity of a factor of two was observed at the width of approximately 15 nm for the <111> orientation compared with other orientations. By contrast, polycrystalline W nanowires (serving as control samples) did not exhibit anisotropy. This result, which is not limited to just W, indicates the critical role of crystallographic orientation in nanoscale metallic conduction.

## Results

### Properties of W films and nanowires

Nominally 20 nm-thick, (110) epitaxial W and polycrystalline W films were prepared on 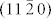-Al_2_O_3_ and thermally oxidized (100)-silicon, respectively. Table I lists the crystal orientation, thickness and the resistivity of the films. Table I also gives the grain size for the polycrystalline film. (See Methods and Supplementary Fig. S1–Fig. S4 for details of the film preparation and characterization.)

**Figure 1:**
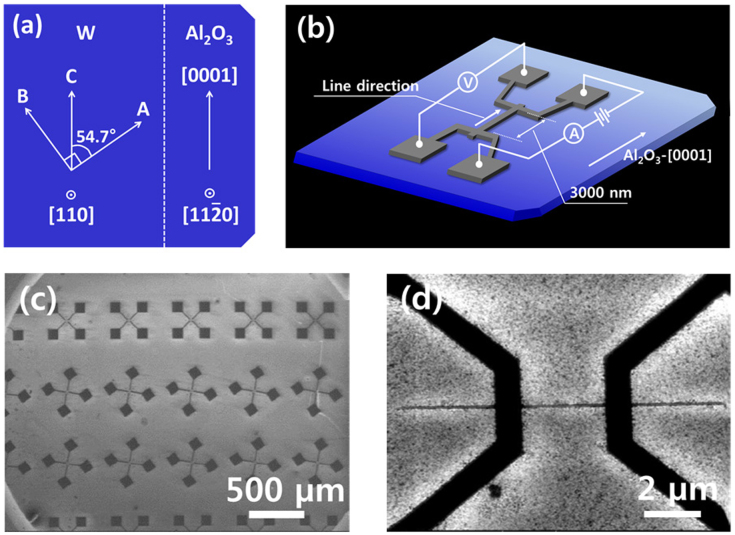
(a) (Top-view) Crystallographic orientations for the epitaxial (110)-W film (on the left) and the (11
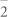
0)-Al_2_O_3_ substrate (on the right) are presented. Directions of A, B and C correspond to the [001], 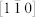 and 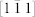 orientations of bcc-W (See Table II), (b) The schematic patterned structure with the electrical measurement set-up. The wire direction in this particular schematic corresponds to the C orientation. (c) A low-magnification scanning electron microscopy image (SEM) that shows the patterned structure with the three wire directions. The W wires in the first, second and third horizontal rows from the top correspond to the A, B and C directions, respectively. (d) A high-magnification SEM image that shows a single W wire.

Single crystal W nanowires with longitudinal orientation along the <100>, <110> and <111> directions of bcc W were formed by subtractively patterning the epitaxial W film with reactive ion etching (RIE) (See Supplementary Fig. S5 for the fabrication processes for the nanowires and Supplementary Fig. S3 for the determination of the crystallographic orientations of the nanowires.) The relevant crystallographic orientations for the W film and the Al_2_O_3_ substrate, including the three wire directions, are summarized in Fig. 1(a). Wire-widths were in the range of 14.8–451.3 nm and are given in Table II. The nanowire lengths were fixed at 3 μm. To provide control samples, nanowires were formed on the polycrystalline W film by applying identical fabrication conditions, including the angular distribution of the three wire directions. Figure 1(b) shows the schematic of the patterned structure with the electrical measurement configuration. Nanowire width and resistivity were determined using the temperature coefficient of resistance (TCR) method. [See Supplementary Discussion] Scanning electron microscopy (SEM) images of the patterned structures are given in Figs. 1(c) and (d).

### Anisotropy of the resistivity increase with decreasing width for the single crystal W nanowires

**Figure 2:**
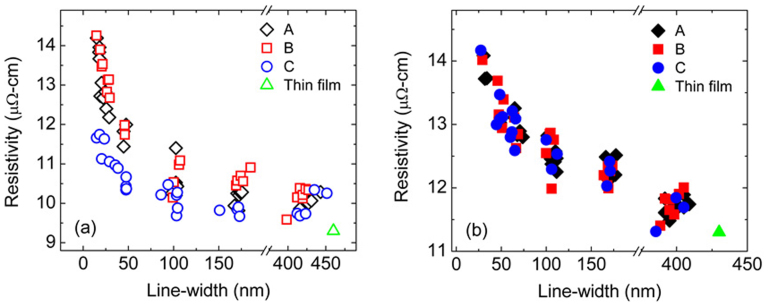
(a) Resistivity as a function of wire-width for (a) the single crystal wires and (b) the polycrystalline wires in the A, B and C orientations. The thin film resistivities prior to patterning are also plotted.

Figure 2(a) plots wire resistivities at 298 K as a function of width for the single crystal nanowires having wire orientations in the <100>, <110> and <111> directions of bcc W. The resistivities are in the range of 9.6 to 14.3 μΩ-cm. A clear anisotropy is seen for nanowire widths below 50 nm, with the smallest size effect in the <111> direction (Fig. 2a). The anisotropy of the resistivity becomes less pronounced with increasing nanowire width and eventually disappears as the wire resistivity approaches the film resistivity. By contrast, the polycrystalline nanowires (control samples) do not exhibit anisotropy in the measured resistivity, as shown in Fig. 2(b). Resistivities for both the single crystal and polycrystalline nanowires at 150 K show a similar trend. Table II summarizes the widths and resistivities for the narrowest and widest wires for the three orientations of the single crystal and the polycrystalline nanowires. It is worth noting that the resistivities of 9.6 and 11.4 *μ*Ω-cm, obtained for the widest single crystal and polycrystalline wires, are close to the corresponding blanket thin film resistivities of 9.3 and 11.3 *μ*Ω-cm, respectively.

## Discussion

The Fermi surface of W is composed of four closed parts: the hole octahedron, hole ellipsoid, electron jack and electron ball^12^,^13^. The complex shape of the Fermi surface reflects a significant anisotropy associated with the conduction electrons in W^14^. This anisotropy is most pronounced for the Fermi velocity of the hole octahedron, with values of 5.3, 9.5 and 15.5 × 10^6^ ms^−1^ in the <100>, <110> and <111> directions, respectively^15^. By contrast, a significantly lower anisotropy is associated with the other three parts of the Fermi surface^14^. Table III summarizes reported values of the area fractions, ranges of the Fermi velocity, and anisotropy (defined as the ratio of maximum to minimum Fermi velocity) for the four components of the Fermi surface.

Since the dominant scattering mechanism in sufficiently thin single crystal films and sufficiently narrow single crystal nanowires giving rise to the size effect is the non-specular scattering of electrons from the conductor's surfaces, it is predicted that the anisotropic size effect in W nanowires is related to the non-symmetric distribution of electron momentum (or velocity). The hole octahedron, with its pronounced anisotropy of Fermi velocity (factor of 2.9 compared to 1.1–1.6 for the other three parts of the Fermi surface) and its relatively large share of the Fermi surface (33%), is therefore expected to play a dominant role in the anisotropy of the size effect.

**Figure 3:**
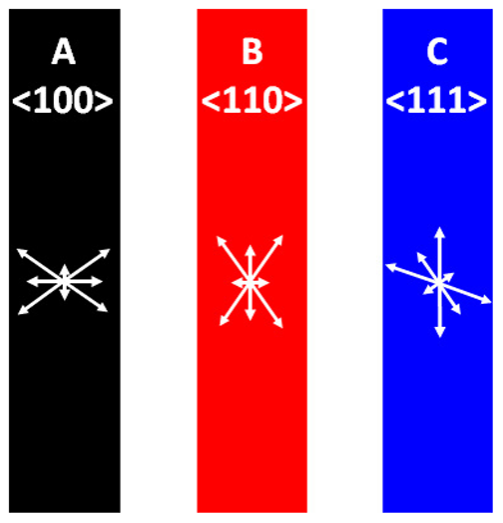
Illustration of the single crystal wires laid parallel to the <100>, <110> and <111> orientations. The arrows represent the Fermi velocities for the hole octahedron in the <100>, <110> and <111> orientations. The arrows are drawn following the vector system (i.e., the directions and lengths of the arrows represent the orientations and magnitudes of the Fermi velocity).

Figure 3 gives a two-dimensional geometric relation between the orientation of the single crystal wires identified in Fig. 1(a) and the Fermi velocities (following the vector notation) for the hole octahedron in these orientations. Ziman reported that relaxation times for phonon and impurity scatterings are almost isotropic for the “belly” and “neck” electrons in Cu near its Debye temperature (343.5 K)^16^. If W exhibits a similar isotropic behavior of relaxation times, the Fermi velocity distribution given in Fig. 3 can be also considered to be the relative distribution of the electron mean free path in different crystal directions. Based on this, the observed resistivity size effect in W nanowires can be explained by focusing on the largest (and most influential) Fermi velocity relative to the sidewalls of the wires. For the wires in the <100> and <110> orientations, the two largest components of the Fermi velocity are directed at the surfaces at 54.7° and 35.3° with respect to the wire directions. When the electrons with these Fermi velocities experience non-specular surface scattering, the loss of electron momentum along the wire axis, i.e., the cosine contribution, is relatively large, giving rise to a steep resistivity size effect. By contrast, the wires with the <111> orientation have their two largest Fermi velocity components at 0° and 70.5° with respect to the wire direction. The former component, being parallel with the wire surfaces, is not expected to be affected by surface scattering, and the cosine contribution of the latter component is relatively small when compared with the <100> and <110>-oriented wires. This geometric argument gives a simple, but compelling description for the significantly smaller resistivity increase for the nanowires having the <111> orientation. For the polycrystalline nanowire where there are some 30 grains with random in-plane orientations along the nanowires, the anisotropy is expected to be averaged out, which is in agreement with Fig. 2 (b).

**Figure 4:**
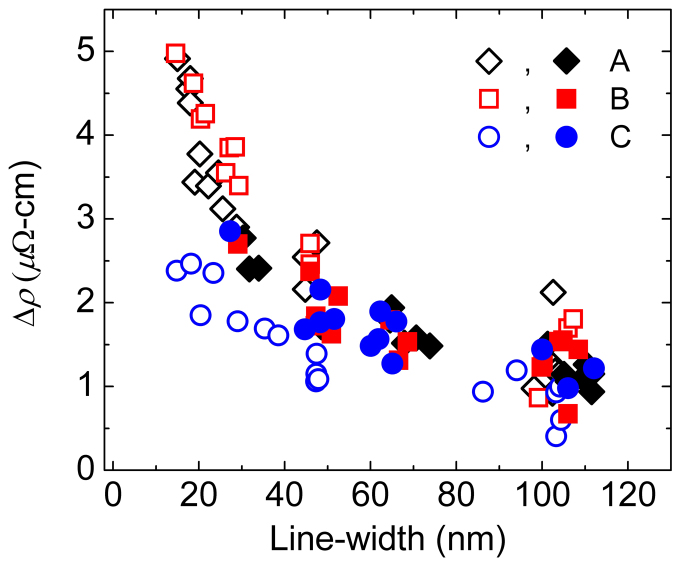
Increase in resistivity with respect to the thin film resistivity is plotted as a function of wire-width for the single crystal (open symbols) and polycrystalline (closed symbols) wires for the three directions given in Table II.

The increase in resistivities for the single crystal and polycrystalline nanowires relative to the thin film resistivities are plotted together in Fig. 4 as a function of nanowire-width. They show an increase of 2.4 *μ*Ω-cm for the 14.8 nm-wide <111> oriented nanowire as compared with an increase of 4.9 and 5.0 *μ*Ω-cm for the 15.0 nm wide <100> and 14.6 nm wide <110> oriented nanowires, respectively. For the polycrystalline nanowires, the incremental increase in resistivity at the nanowire-width of 29.1 nm is 2.7 *μ*Ω-cm, as compared with 1.8 *μ*Ω-cm for the <111> oriented single crystal nanowires at a nearly similar wire-width of 29.0 nm. Furthermore, Fig. 4 implies that the contribution of surface scattering to the resistivity size effect for the polycrystalline nanowires is higher than the smallest (i.e., the <111>-nanowires) but lower than the largest (i.e., the <110>-nanowires) resistivity size effect seen in the single crystal nanowires. We also note that since the grain size of the polycrystalline W nanowires is determined by the grain size of the polycrystalline film and is thus independent of wire-width, the contribution of grain boundary scattering to the resistivity of the polycrystalline nanowires should be independent of wire-width so long as Mathiessen's rule regarding the independent contributions of surface and grain boundary scattering holds for W as it does for Cu^17^,^18^. Therefore, Fig. 4 for the polycrystalline nanowires gives only the contribution to the resistivity increase from surface scattering.

In conclusion, we demonstrate an anisotropy in the resistivity size effect, using single crystal body centered cubic tungsten nanowires with their longitudinal orientations along the <100>, <110> and <111> crystallographic directions. The anisotropy is observed for wire-widths below 50 nm and the smallest increase in resistivity relative to an infinitely wide wire is observed for nanowires having the <111> orientations. The anisotropy of the size effect is attributed to the geometric relation between the crystallographic orientation of the wire and the anisotropic Fermi velocity for the hole octahedron, which leads to anisotropic momentum loss along the length of the single crystal nanowires.

## Methods

### Preparation of the single crystal and polycrystalline W films

A (110)-epitaxial W film and a polycrystalline W film were deposited at 520°C by DC magnetron sputtering using a 99.95% pure W target. The substrate for the epitaxial film was (11
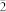
0)-Al_2_O_3_, whereas a thermally oxidized (100)-Si substrate was used for the polycrystalline film. Sputtering power was fixed at 250 W. The deposition rate at this power was 1.4 Å/sec. Following deposition, the films were annealed at 850°C for two hours in Ar + 4% H_2_ ambient^19^.

### Characterization of the single crystal and polycrystalline W films

The X-ray diffraction (XRD) analyses and determination of the crystallographic orientations for the single crystal film are given in Supplementary Figs. S1 and. S3, whereas Supplementary Figs. S2 and S4 provide XRD and dark field transmission electron micrograph (TEM) analyses for the polycrystalline W film. The polycrystalline film was found to be <110> fiber-textured by an X-ray *ψ*-scan, i.e., where the sample was tilted about an axis in the scattering plane, perpendicular to the scattering vector. The mean in-plane grain size of the polycrystalline film is ~100 nm, estimated by plan-view dark field transmission electron micrographs. [See Supplementary Fig. S4] Film thicknesses were measured by X-ray reflectivity. Film resistivities were measured using the van der Pauw method^12^,^19^,^20^.

### Formation of the W nanowires

Single crystal W nanowires with the longitudinal orientations along the <100>, <110> and <111> directions of bcc W were formed by subtractively patterning the epitaxial W film with reactive ion etching (RIE) using a mixture of SF_6_, CHF_3_ and O_2_. A combination of patterned electron beam resist (hydrogen silsesquioxane) and photoresist (AZ4110) was used as etch masks for pattern transfer. (See Supplementary Fig. S5 for the detail process flow for the nanowire formation).

## Author Contributions

The idea and experiments in this study were conceived by D.C., K.R.C. and K.B. D.C. prepared the single crystal and polycrystalline films and characterized them. D.C. and M.M. fabricated W nanowires. X.L. measured the grain size of the polycrystalline W film. D.C., S.J.O. and C.R.K. were involved in the resistivity measurement for the W nanowires. D.C., K.R.C. and K.B. analyzed the results and wrote the manuscript. All authors commented on the final manuscript.

## Supplementary Material

Supplementary Information Supplementary Information

## Figures and Tables

**Table 1:** Film type, film normal orientation, thickness, resistivity, and grain size are given

Type	Orientation	Thickness (nm)	Resistivity (μΩ-cm)	Grain size (nm)
Epitaxial	(110)	21.2	9.3	-
Polycrystalline	<110> fiber-texture	20.5	11.3	100

**Table 2:** Wire direction identification, corresponding crystallographic wire directions in bcc W, narrowest wire-widths and wire resistivities, and widest wire-widths and wire resistivities are given. The wire-length is fixed at 3 μm for all the wires

Wire i.d.	Wire direction	Narrowest wire-width (nm)	Wire resistivity (*μ*Ω-cm)	Widest wire-width (nm)	Wire resistivity (*μ*Ω-cm)
A	<100>	15.0	14.2	442.9	9.8
B	<110>	14.6	14.3	427.0	9.6
C	<111>	14.8	11.7	451.3	9.7
A,B,C	Polycrystalline	27.3	14.2	408.9	11.4

**Table 3:** Parts comprising the Fermi surface of W, their surface area fractions, Fermi velocity and anisotropy are given. Anisotropy is defined as the ratio of maximum to minimum Fermi velocity

Parts	Surface area (%)^21^	Fermi velocity (10^5^ m/sec)^14^,^15^	Anisotropy
Hole octahedron	33	5.3–15.5	2.9
Hole ellipsoid	12	5.7–6.2	1.1
Electron jack	55	7.5–9.2	1.2
Electron ball		4.2–6.7	1.6
