# Supplementary material for: Crystallographic anisotropy of the resistivity size effect in single crystal tungsten nanowires
Source: Sci Rep. 2013 Sep 5;3:2591. doi: 10.1038/srep02591 (PMC3763248; doi:10.1038/srep02591)
Supplement: Supplementary Information [file srep02591-s1.pdf]

Supplementary Information

Crystallographic anisotropy of the resistivity size effect in single crystal tungsten nanowires

Dooho Choi[1], Matthew Moneck[2], Xuan Liu[1], Soong Ju Oh[3], Cherie R. Kagan[3,4], Kevin R. Coffey[5] and Katayun Barmak[1,6]

[1]Department of Materials Science and Engineering, Carnegie Mellon University, 5000 Forbes Avenue, Pittsburgh, Pennsylvania 15213, USA

[2]Department of Electrical and Computer Engineering, Carnegie Mellon University, 5000 Forbes Avenue, Pittsburgh, Pennsylvania 15213, USA

[3]Department of Materials Science and Engineering, University of Pennsylvania, 3451 Walnut Street, Philadelphia, Pennsylvania 19104, USA

[4]Department of Electrical and Systems Engineering, University of Pennsylvania, 3451 Walnut Street, Philadelphia, Pennsylvania 19104, USA

[5]Department of Materials Science and Engineering, University of Central Florida, 4000 Central Florida Boulevard, Orlando, Florida 32816, USA

[6]Department of Applied Physics and Applied Mathematics, Columbia University, 500 West 120th Street, New York, New York 10027, USA

Supplementary figures

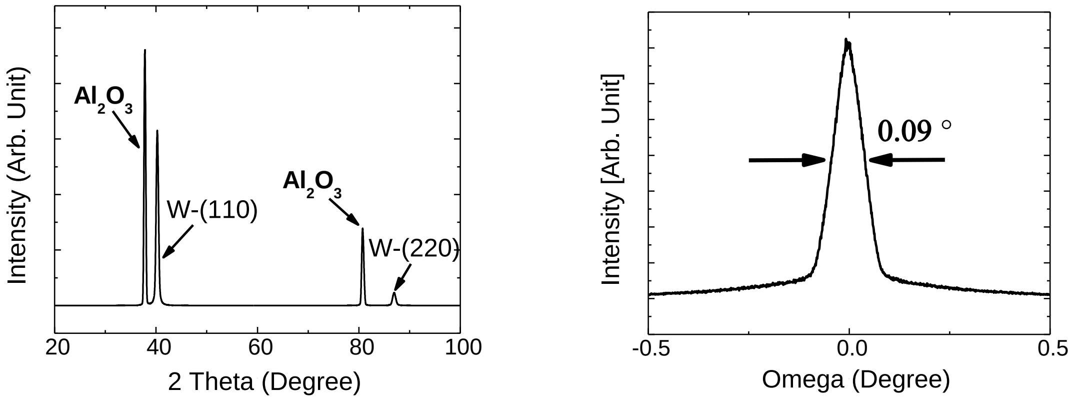

Supplementary Figure S1: X-ray diffraction (XRD) analyses for the single crystal W film. Left panel: θ-2θ XRD pattern is shown. The substrate peaks, from left to right, are $(11\bar{2}0)$ and $(22\bar{4}0)$, respectively. Right panel: Rocking curve (ω scan) with respect to the W-(110) peak. The full width half maximum (FWHM) value was 0.09°.

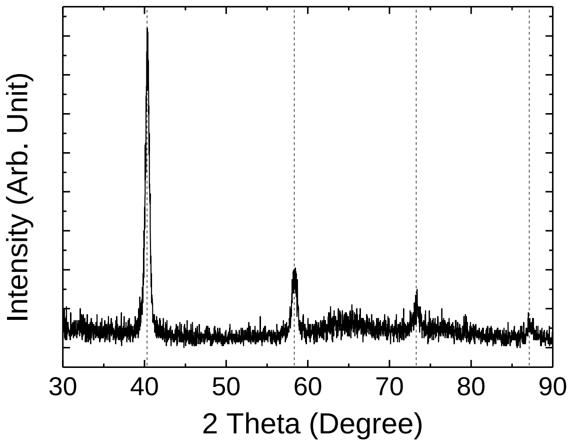

Supplementary Figure S2: *θ*-2*θ* XRD scan for the polycrystalline W film. The solid and dotted vertical lines mark the positions of the Bragg peaks for bcc W. The four Bragg peaks, from left to right, are (110), (200), (211) and (220) for bcc W. No high-resistivity A15 *β*-phase was observed. The sample was omega-tilted by 6.5° to suppress the strong Si-(400) substrate peak near 69.2°, without significantly affecting the intensity of the W peaks.

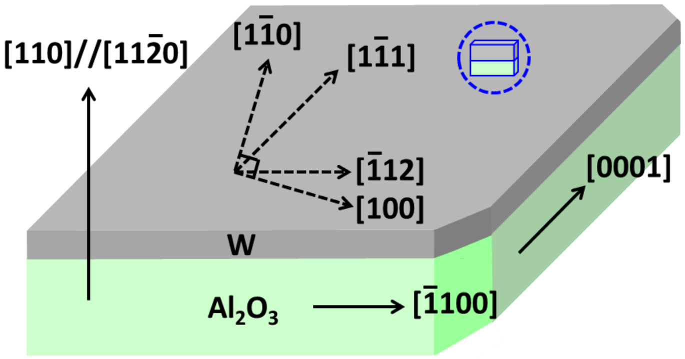

Supplementary Figure S3: Determination of the crystallographic orientations. The Al_2O_3 substrate received from MTI Corporation was beveled at two corners as schematically drawn above to clearly mark the side parallel to the <0001> direction. The cross-sectional TEM specimen was prepared perpendicular to this side using a focused ion beam (FIB) system. (see the structure in the blue circle) Relevant crystallographic orientations for W and Al_2O_3 are presented in the figure. The dotted arrows represent in-plane orientations for the W film. The high resolution cross-sectional TEM image provided elsewhere[1] shows that the measured interplanar spacing along the film normal is very close to the value of d_{110} for W, which is in agreement with the result in Supplementary Figure S1. The Fourier-transformed lattice images for the W film and Al_2O_3 substrate acquired from the cross-section give [1$\bar{1}$1] zone axis pattern for W and [0001] zone axis pattern for Al_2O_3, which are consistent with the directions provided in the schematic.

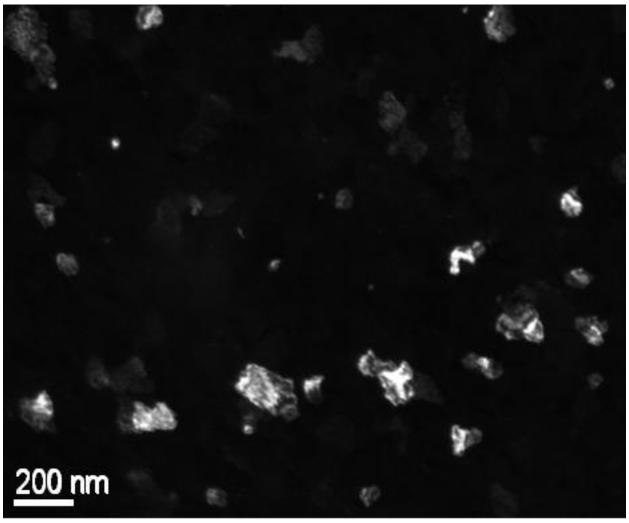

Supplementary Figure S4: Dark-field transmission electron micrograph for the polycrystalline W film. The image was formed using the (110) reflected beam. The in-plane grain size is approximately 100 nm.

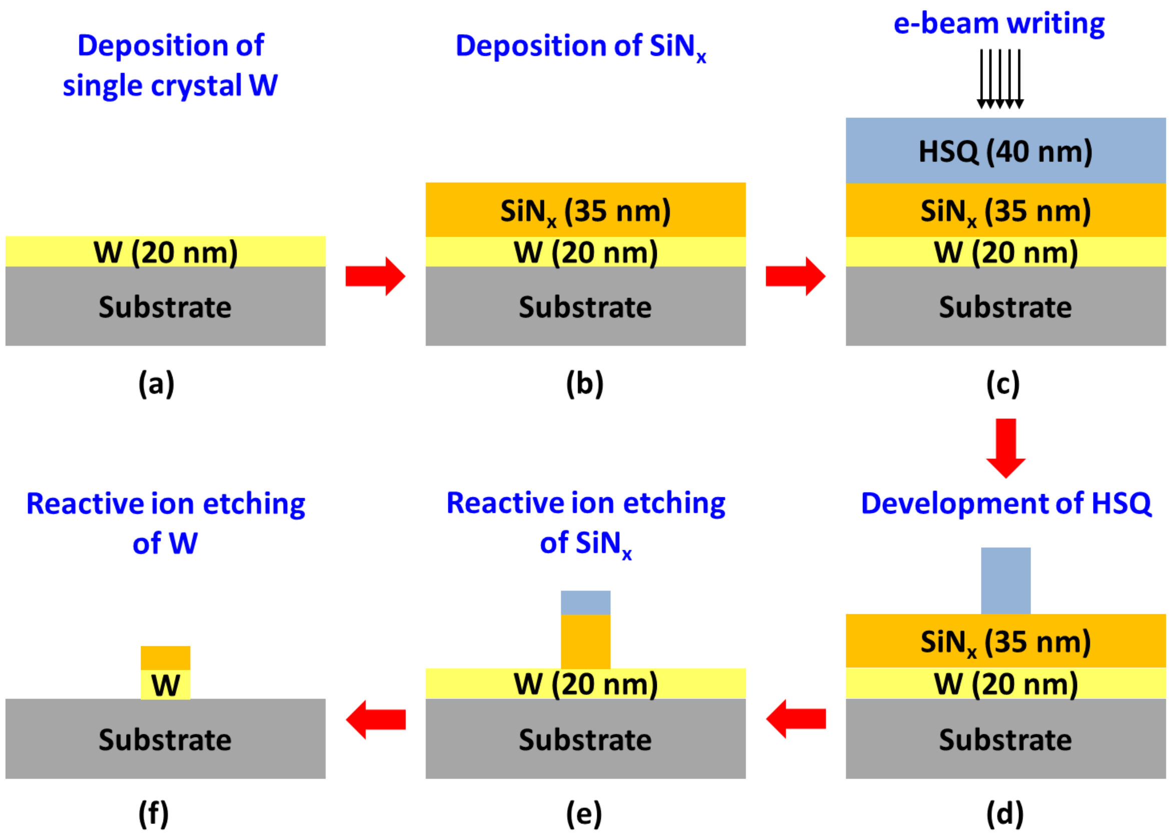

Supplementary Figure S5: Process flow for the nanowire formation. (a) Nominally 20 nm-thick epitaxial and polycrystalline W films were deposited. (b) A 35 nm-thick SiN_x hard mask was deposited by reactive sputtering from a Si_3N_4 target in order to serve as a hard mask and promote the adhesion of hydrogen silsesquioxane (HSQ). Base pressure, working pressure, power and dc bias for the SiN_x layer deposition were 1×10^{-7} Torr, 5 mTorr, 300 W and -1790 V, respectively. Flow rates of Ar and N_2 were 18 sccm and 9 sccm, respectively, corresponding to respective partial pressures of 3.5 mTorr and 1.5 mTorr. (c) Dow Corning XR1541 electron beam resist consisting of 2% HSQ diluted in methyl isobutyl ketone (MIBK) was spincoated onto the SiN_x layer with a dynamic dispense at 600 rpm for 6 seconds, followed by spinning at 2000 rpm for 60 seconds. After the spin coating process, the samples were baked on a hot plate for 1 minute at 180 ℃. Electron beam (e-beam) writing of the nanowires was performed in a FEI Sirion SEM running J.C. Nabity Nanometer Pattern Generation System (NPGS) software. The e-beam acceleration voltage, beam current, and dosage were 30 kV, 32

pA and 400-450 $\mu C/cm^2$, respectively. (d) Ultrasonic development was performed in Microposit MF-CD26, a 0.26 N Tetramethylammonium hydroxide (TMAH) based developer, for 36 sec, followed by a DI water rinse and a N_2 drying step. (e) The SiN_x layer was etched by reactive ion etching (RIE), for which RF power, DC bias, base pressure, working pressure were 25 W, -225 V, 10^{-5} Torr and 10 mTorr, respectively. Following the RIE transfer of the nanowire pattern into the SiN_x, AZ4110 photoresist (not shown here) was spincoated onto the sample with a static dispense followed by a 600 rpm, 6 sec spread and a 4000 rpm, 60 sec spin. After spin coating, the samples were baked for 5 min on a hotplate at 95° C. The nanowire leads and probe pad patterns were exposed in the photoresist using contact lithography with a borosilicate mask and a mercury arc lamp. The exposure was 60 sec long at a wavelength of 312 nm and a power density of ~5mW/cm^2. The photoresist was developed for 2 min in AZ Developer solution mixed 2:1 by volume with DI water. (f) Finally, W was etched by RIE (see Supplementary Table S1 for details) with the patterned SiN_x acting as a hard mask for the nanowires and the patterned photoresist acting as a mask for the leads and probe pads.

Supplementary Table

Supplementary Table S1: Reactive ion etching (RIE) process for the W nanowire formation. Detailed etching conditions along with etch rates for the polycrystalline W film, single crystal W film and SiNx film are given.

Gas flow rate (sccm)			Base pressure (Torr)	Working pressure (mTorr)	Power (W)	DC bias (V)	Etch rate (nm/min)		
SF_6	CH_3	O_2	10^{-6}	45	30	-100	Poly-crystalline W	Single crystal W	SiN_x
5	5.8	1.9					50	30	27

Supplementary Discussion

Line-width measurement using the temperature coefficient of resistance (TCR) method.

In order to measure electrical resistivity of a nanowire, the cross-sectional area of the nanowire must be measured. The top surface of the W nanowires is protected by a SiN_x hard mask (see Figure S4) allowing the line-height to remain intact during the patterning processes, but the line-widths are prone to local variations of the processing conditions, which inevitably result in non-uniform widths. To circumvent the practical difficulties in measuring the non-uniform line dimensions, an effective, or average, cross-sectional area of each nanowire was measured using the "temperature coefficient of resistance (TCR)" method,[2] as described below.

The electrical resistance of a nanowire is

$$R = \rho \times \frac{l}{w \times h}. \quad \text{(S1)}$$

where R and ρ are resistance and resistivity, and l, w and h are length, width and height for a nanowire. The resistance difference of a nanowire at two different temperatures can be computed using the relation,

$$R_2 - R_1 = (\rho_2 - \rho_1) \times \frac{l}{w \times h}. \quad \text{(S2)}$$

According to Matthiessen's rule, the total resistivity of a structure is the sum of the temperature-dependent term (i.e., phonon contribution) and the temperature-independent term (i.e., static defect contribution).[2]

$$\rho_{total}(T) = \rho_{phonon}(T) + \rho_{defects}. \quad \text{(S3)}$$

The derivative of the total film resistivity then becomes equal to the derivative of the phonon term only, i.e.,

$$\frac{d\left(\rho_{total}(T)\right)}{dT} = \frac{d\left(\rho_{phonon}(T)\right)}{dT}. \qquad \text{(S4)}$$

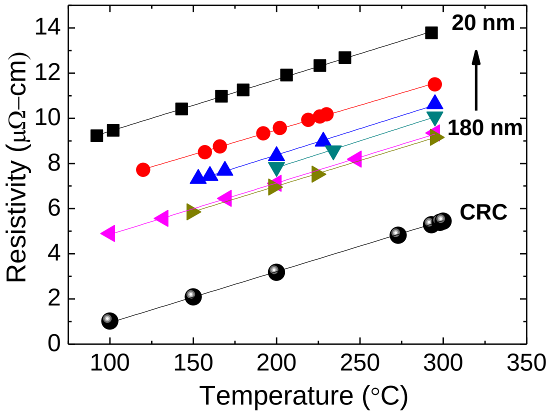

Thickness (nm)	$\frac{d\rho}{dT}$	R^2
20	0.023	0.9995
30	0.023	0.9992
40	0.022	0.9989
60	0.022	0.9989
120	0.023	0.9996
180	0.022	0.9996
CRC data	0.023	0.9994

The figure above shows resistivity as a function of temperature for a set of polycrystalline W films with thickness ranging from 20 nm and 180 nm prepared in our laboratory. The resistivity vs. temperature values for a pure, single crystal, bulk W sample from a *CRC handbook* are also plotted in the figure.[3] The higher resistivities for the thinner films are due to the more frequent electron scattering at film surfaces. It is noteworthy that the bulk resistivity for our films (i.e., the resistivity for the 180-nm thick film) is 9.2 $\mu\Omega$-cm at room temperature, which is significantly higher than the value of 5.3 $\mu\Omega$-cm for W in the *CRC* handbook. The higher resistivity is a result of the shortened electron mean free path created by the additional electron scattering at static defects, such as impurities. The extracted values of $\left(\frac{d\rho}{dT}\right)$ from the linear fits and the corresponding R^2 values of the method of least squares are listed in table above. The very similar values of $\left(\frac{d\rho}{dT}\right)$ and the R^2 values being very close to 1 for all the fits suggest that the use of Eq. (S4) for the single crystal W nanowires in this study, with their similar dimensions to the thin films in table above, is appropriate.

The linear resistivity-temperature relation allows Eq. (S2) to be rewritten as

$$R_2 - R_1 = \left(\frac{d\rho}{dT}\right) \times (T_2 - T_1) \times \frac{l}{w \times h}. \quad \text{(S5)}$$

Using the measured resistances at two temperatures (298 and 150 K), the value of 0.023 for $\left(\frac{d\rho}{dT}\right)$, and a value of 3000 nm for the line-length, the cross-sectional area ($w \times h$) of a nanowire can be computed, which, in turn, allows the resistivity at the particular cross-sectional area to be computed using Eq. (S1).

Supplementary References

1. D. Choi, C. S. Kim, D. N. S. Chung, A. P. Warren, N. T. Nuhfer, M. F. Toney, K. R. Coffey and K. Barmak, Phys. Rev. B 045432 (2012).

2. W. Steinhögl, G. Schindler, G. Steinlesberger, M. Traving, and M. Engelhardt Steinhogel, J. Appl. Phys. **97**, 023706 (2005)

3. CRC Handbook of Chemistry and Physics, 92th ed. (Taylor & Francis, London, 2012), p. 12-41 [http://www.hbcpnetbase.com/]
